# Supplementary material for: The effect of menopause on cardiovascular risk factors according to body mass index in middle-aged Korean women
Source: PLoS One. 2023 Mar 23;18(3):e0283393. doi: 10.1371/journal.pone.0283393 (PMC10035845; doi:10.1371/journal.pone.0283393)
Supplement: Attachment — Submitted filename: PONE-D-22-33212 reviewer comments.pdf [file pone.0283393.s001.pdf]

PLOS ONE

The effect of menopause on cardiovascular risk factors according to body mass index in middle-aged Korean women

--Manuscript Draft--

**Manuscript Number:**	PONE-D-22-33212
**Article Type:**	Research Article
**Full Title:**	The effect of menopause on cardiovascular risk factors according to body mass index in middle-aged Korean women
**Short Title:**	Menopause and cardiovascular risk factors
**Corresponding Author:**	Hyejin Lee<br>Ewha Womans University School of Medicine<br>Seoul, KOREA, REPUBLIC OF
**Keywords:**	menopause, body mass index, cardiovascular diseases
**Abstract:**	Background: Menopausal status and obesity are associated with increased risk for cardiovascular diseases. However, there are few studies on the effect of menopause on cardiovascular risk factors according to the degree of obesity during the menopausal transition. We aimed to evaluate the effect of menopause on cardiovascular risk factors according to body mass index (BMI) in middle-aged Korean women.<br>Methods: We analyzed 361 postmenopausal women and 758 premenopausal women (age: 45-55 years) without diabetes mellitus, hypertension, or dyslipidemia, using a cohort database released by the Korean National Health and Nutrition Examination Survey 2016-2018. Subjects were divided into two groups based on BMI. Women who underwent a hysterectomy or were pregnant were excluded from this study.<br>Results: Postmenopausal women were older than premenopausal women, and BMI did not differ between the two groups. After adjustment for age and BMI in total and non-obese subjects (not obese subjects), postmenopausal women exhibited higher hemoglobin A1c and total cholesterol levels than premenopausal women. Subgroup analysis for 138 postmenopausal and 138 age- and BMI-matched premenopausal women showed that postmenopausal women had higher total cholesterol levels than premenopausal women with marginal significance.<br>Conclusion: Menopausal status was associated with glucose and cholesterol levels independent of age and BMI in middle-aged Korean women. Menopausal status showed a significant relationship with increased total cholesterol levels even after adjusting for age and BMI in non-obese women but not in obese women. Therefore, intensive monitoring and treating lipid status is necessary to prevent cardiovascular events during the transition to menopause, especially in non-obese subjects.
**Order of Authors:**	Do Kyeong Song, M.D.
	Young Sun Hong
	Yeon-Ah Sung
	Hyejin Lee
**Additional Information:**	
**Question**	**Response**
**Financial Disclosure**<br><br>Enter a financial disclosure statement that describes the sources of funding for the work included in this submission. Review the submission guidelines for detailed requirements. View published research articles from *PLOS ONE* for specific	The author(s) received no specific funding for this work.

examples.<br><br>This statement is required for submission and **will appear in the published article** if the submission is accepted. Please make sure it is accurate.<br><br>**Unfunded studies**<br>Enter: *The author(s) received no specific funding for this work.*<br><br>**Funded studies**<br>Enter a statement with the following details:<br>• Initials of the authors who received each award<br>• Grant numbers awarded to each author<br>• The full name of each funder<br>• URL of each funder website<br>• Did the sponsors or funders play any role in the study design, data collection and analysis, decision to publish, or preparation of the manuscript?<br>• **NO** - Include this sentence at the end of your statement: *The funders had no role in study design, data collection and analysis, decision to publish, or preparation of the manuscript.*<br>• **YES** - Specify the role(s) played.<br><br>* typeset	
**Competing Interests**<br><br>Use the instructions below to enter a competing interest statement for this submission. On behalf of all authors, disclose any competing interests that could be perceived to bias this work—acknowledging all financial support and any other relevant financial or non-financial competing interests.<br><br>This statement is required for submission and **will appear in the published article** if the submission is accepted. Please make sure it is accurate and that any funding sources listed in your Funding Information later in the submission form are also declared in your Financial Disclosure statement.	The authors have declared that no competing interests exist.

View published research articles from *PLOS ONE* for specific examples.<br><br>**NO authors have competing interests**<br><br>Enter: *The authors have declared that no competing interests exist.*<br><br>**Authors with competing interests**<br><br>Enter competing interest details beginning with this statement:<br><br>*I have read the journal's policy and the authors of this manuscript have the following competing interests: [insert competing interests here]*<br><br>* typeset	
**Ethics Statement**<br><br>Enter an ethics statement for this submission. This statement is required if the study involved:<br><br>• Human participants<br>• Human specimens or tissue<br>• Vertebrate animals or cephalopods<br>• Vertebrate embryos or tissues<br>• Field research<br><br>Write "N/A" if the submission does not require an ethics statement.<br><br>General guidance is provided below. Consult the submission guidelines for detailed instructions. **Make sure that all information entered here is included in the Methods section of the manuscript.**	This study was approved by the Institutional Review Board of Ewha Medical Center. We did not obtain informed consent from individuals because data were not collected for the study.

Powered by Editorial Manager® and ProduXion Manager® from Aries Systems Corporation

**Format for specific study types**<br><br>**Human Subject Research (involving human participants and/or tissue)**<br>• Give the name of the institutional review board or ethics committee that approved the study<br>• Include the approval number and/or a statement indicating approval of this research<br>• Indicate the form of consent obtained (written/oral) or the reason that consent was not obtained (e.g. the data were analyzed anonymously)<br><br>**Animal Research (involving vertebrate animals, embryos or tissues)**<br>• Provide the name of the Institutional Animal Care and Use Committee (IACUC) or other relevant ethics board that reviewed the study protocol, and indicate whether they approved this research or granted a formal waiver of ethical approval<br>• Include an approval number if one was obtained<br>• If the study involved *non-human primates*, add *additional details* about animal welfare and steps taken to ameliorate suffering<br>• If anesthesia, euthanasia, or any kind of animal sacrifice is part of the study, include briefly which substances and/or methods were applied<br><br>**Field Research**<br><br>Include the following details if this study involves the collection of plant, animal, or other materials from a natural setting:<br>• Field permit number<br>• Name of the institution or relevant body that granted permission	
**Data Availability**<br><br>Authors are required to make all data underlying the findings described fully available, without restriction, and from the time of publication. PLOS allows rare exceptions to address legal and ethical concerns. See the PLOS Data Policy and FAQ for detailed information.	Yes - all data are fully available without restriction

A Data Availability Statement describing where the data can be found is required at submission. Your answers to this question constitute the Data Availability Statement and **will be published in the article**, if accepted.<br><br>**Important:** Stating 'data available on request from the author' is not sufficient. If your data are only available upon request, select 'No' for the first question and explain your exceptional situation in the text box.<br><br>Do the authors confirm that all data underlying the findings described in their manuscript are fully available without restriction?	
**Describe where the data may be found in full sentences. If you are copying our sample text, replace any instances of XXX with the appropriate details.**<br><br>• If the data are **held or will be held in a public repository**, include URLs, accession numbers or DOIs. If this information will only be available after acceptance, indicate this by ticking the box below. For example: *All XXX files are available from the XXX database (accession number(s) XXX, XXX.*).<br>• If the data are all contained **within the manuscript and/or Supporting Information files**, enter the following: *All relevant data are within the manuscript and its Supporting Information files.*<br>• If neither of these applies but you are able to provide **details of access elsewhere**, with or without limitations, please do so. For example:<br><br>*Data cannot be shared publicly because of [XXX]. Data are available from the XXX Institutional Data Access / Ethics Committee (contact via XXX) for researchers who meet the criteria for access to confidential data.*<br><br>*The data underlying the results presented in the study are available from (include the name of the third party*	All relevant data are within the manuscript

Powered by Editorial Manager® and ProduXion Manager® from Aries Systems Corporation

and contact information or URL).<br>• This text is appropriate if the data are owned by a third party and authors do not have permission to share the data.<br><br>* typeset	
Additional data availability information:	

Manuscript

Click here to access/download;Manuscript;Menopause PLOS ONE.docx

The effect of menopause on cardiovascular risk factors according to body mass index in middle-aged Korean women

Short title: Menopause and cardiovascular risk factors

Do Kyeong Song, Young Sun Hong, Yeon-Ah Sung, Hyejin Lee*

Department of Internal Medicine,

Ewha Womans University School of Medicine, Seoul, Korea

* **Correspondence:** Hyejin Lee, M.D., Ph.D.

E-mail: hyejinlee@ewha.ac.kr (HL)

Abstract

Background: Menopausal status and obesity are associated with increased risk for cardiovascular diseases. However, there are few studies on the effect of menopause on cardiovascular risk factors according to the degree of obesity during the menopausal transition. We aimed to evaluate the effect of menopause on cardiovascular risk factors according to body mass index (BMI) in middle-aged Korean women.

Methods: We analyzed 361 postmenopausal women and 758 premenopausal women (age: 45-55 years) without diabetes mellitus, hypertension, or dyslipidemia, using a cohort database released by the Korean National Health and Nutrition Examination Survey 2016-2018. Subjects were divided into two groups based on BMI. Women who underwent a hysterectomy or were pregnant were excluded from this study.

Results: Postmenopausal women were older than premenopausal women, and BMI did not differ between the two groups. After adjustment for age and BMI in total and non-obese subjects (not obese subjects), postmenopausal women exhibited higher hemoglobin A1c and total cholesterol levels than premenopausal women. Subgroup analysis for 138 postmenopausal and 138 age- and BMI-matched premenopausal women showed that postmenopausal women had higher total cholesterol levels than premenopausal women with marginal significance.

Conclusion: Menopausal status was associated with glucose and cholesterol levels independent of age and BMI in middle-aged Korean women. Menopausal status showed a significant relationship with increased total cholesterol levels even after adjusting for age and BMI in non-obese women but not in obese women. Therefore, intensive monitoring and treating lipid status is necessary to prevent cardiovascular events during the transition to menopause, especially in non-obese subjects.

Introduction

Cardiovascular disease is the leading cause of death for women [1]. Menopausal status is associated with increased risk for cardiovascular diseases mainly due to changes in body fat distribution, glucose metabolism, and serum lipids [2]. Traditionally, menopause is defined as the absence of menstruation with no other cause for 12 consecutive months [3]. The menopausal transition is characterized by ovarian hormone changes, menstrual cycle irregularities, and an increased risk for cardiovascular diseases [4]. Cardiovascular disease incidence rates were higher in postmenopausal women than in premenopausal women in each age group among women below 55 years in a cohort of 2873 Framingham women [5]. A rise in coronary heart disease incidence after menopause was also noted in the Framingham study [6].

However, studies on change in cardiovascular risk factors among women during the menopausal transition demonstrate inconsistent results. Most epidemiological studies show that changes in lipid metabolism during the menopausal transition are associated with a more atherogenic lipid profile, and menopause is associated with increased total cholesterol and low-density lipoprotein (LDL) cholesterol levels. However, there are variations in the lipid profiles during the menopausal transition between studies [7-10]. A cross-sectional study conducted on Korean women (age: 44-56 years), showed that blood pressure was significantly higher during late (than early) menopausal transition [11]. However, blood pressure depended more on age than on the menopausal status among middle-aged Korean women after excluding women on medication for hypertension [8]. It is debatable whether menopause increases cardiovascular risk factors independent of aging. Although the risk of cardiovascular disease linearly increased with increased body mass index (BMI) in premenopausal and postmenopausal Korean women, using a nationwide health examination database [12], the effect of BMI on cardiovascular risk factors during the menopausal transition among women in Korea was unclear.

Furthermore, the effect of postmenopausal status on cardiovascular risk factors is known to vary by ethnicity, partly due to different lifestyles and dietary patterns. Ethnic differences in serum reproductive

hormone concentrations exist independent of menopausal status. Studies show that Chinese and Japanese women exhibit lower estradiol concentrations during the menopausal transition when compared to Caucasian women [13]. To date, there are few studies on the effect of menopause on cardiovascular risk factors according to the degree of obesity during the menopausal transition among women in Korea. We aimed to evaluate the effect of menopause on cardiovascular risk factors according to BMI in middle-aged Korean women.

Materials and Methods

Data source

We used the data obtained from the Korea National Health and Nutrition Examination Survey (KNHANES) from 2016 to 2018. The KNHANES is a national surveillance system assessing the health and nutritional status of non-institutionalized Korean citizens since 1998. This nationally representative cross-sectional survey collects data on socioeconomic status, health-related behaviors, quality of life, healthcare utilization, dietary intakes, anthropometric measures, and biochemical profiles using fasting blood serum and urine and clinical profiles for major chronic diseases through a health interview, nutrition survey, and health examination study annually. The health interview and physical health examination are conducted by trained medical staff and interviewers [14]. Because KNHANES data are nationally representative samples of Korea, including the health interview, physical examination, and nutrition survey, they are important resources to evaluate the relationship between risk factors and diseases in Korea.

We did not obtain informed consent from individuals because data were not collected for the study. The patient records were anonymous before being released by the KNHANES. This study was approved by the Institutional Review Board of Ewha Medical Center. All methods followed the relevant guidelines and regulations.

Study population & outcome variables

We included women (age: 45-55 years), using a cohort database released by the KNHANES 2016-2018. Women who underwent a hysterectomy or were pregnant were excluded from this study. Subjects diagnosed with diabetes, hypertension, or dyslipidemia (based on self-reported questionnaires) were also excluded. Finally, we enrolled 361 postmenopausal and 758 premenopausal women aged 45-55 years.

The BMI was calculated as body weight in kilograms divided by height in meters squared. Body weight and height were measured during the health examinations. Subjects were divided into two groups according to BMI (non-obese subjects: BMI < 25 kg/m^2, obese subjects: BMI ≥ 25 kg/m^2), following Asian-specific criteria [15]. Menopausal status was determined based on self-reported questionnaires; we categorized menopause status as pre- and postmenopausal. Subgroup analysis included 138 postmenopausal women and 138 age- and BMI-matched premenopausal women.

Blood pressure was calculated as the mean of two manual sphygmomanometer readings with the patient in a seated position. A blood sample was obtained in the morning after an overnight fast. Total cholesterol, high-density lipoprotein (HDL) cholesterol, triglycerides, fasting glucose, and hemoglobin A1c (HbA1c) were measured, and LDL cholesterol was calculated using the Friedewald equation [16].

Statistical analysis

The Kolmogorov-Smirnov statistic was used to analyze the continuous variables for normality. Quantitative variables were reported as the means ± the standard deviations. The between-group differences were assessed using the Student unpaired t-tests. Age and BMI were adjusted for analysis of covariance. Multiple linear regression analyses were performed to determine the independent

association between menopausal status and total cholesterol after controlling for age, BMI, systolic blood pressure, and fasting glucose and to determine the association between menopausal status and HbA1c after controlling for age, BMI, systolic blood pressure, and total cholesterol. Statistical analysis was performed using the SPSS 23.0 software package for Windows (IBM Corporation, Chicago, IL, USA). *P* values < 0.05 were considered statistically significant.

Results

Table 1 represents the baseline characteristics of participants by menopausal status. Postmenopausal women were older than premenopausal women (P < 0.05). The mean ages were 48 years for premenopausal women and 52 years for postmenopausal women. BMI did not differ between the two groups. After adjustment for age and BMI, postmenopausal women had higher hemoglobin A1c (HbA1c) and total cholesterol levels than premenopausal women. After adjustment for age and BMI, fasting plasma glucose, HDL cholesterol, triglycerides, LDL cholesterol, systolic blood pressure, and diastolic blood pressure did not differ between postmenopausal and premenopausal women (Table 1).

On dividing the subjects into two groups according to BMI, 33.6% (n = 291) women were postmenopausal in non-obese subjects, and 27.7% (n = 70) women were postmenopausal in obese subjects. Postmenopausal women were older than premenopausal women in non-obese and obese groups (all *P*s < 0.05). After adjustment for age and BMI in non-obese subjects (not obese subjects), postmenopausal women exhibited higher HbA1c and total cholesterol levels than premenopausal women. Fasting glucose, HDL cholesterol, triglycerides, LDL cholesterol, systolic blood pressure, and diastolic blood pressure did not differ between postmenopausal and premenopausal women in non-obese and obese subjects after adjustment for age and BMI (Table 2).

Subgroup analysis for 138 postmenopausal and 138 age- and BMI-matched premenopausal women showed that postmenopausal women had higher total cholesterol levels than premenopausal women

with marginal significance in total (201 ± 25 mg/dL vs. 196 ± 27 mg/dL, P = 0.091) and non-obese subjects (201 ± 25 mg/dL vs. 194 ± 25 mg/dL, P = 0.060) but not in obese subjects (206 ± 23 mg/dL vs. 205 ± 36 mg/dL, P = 0.944). The levels of fasting glucose, HbA1c, HDL cholesterol, triglycerides, LDL cholesterol, systolic blood pressure, and diastolic blood pressure did not differ between postmenopausal and age- and BMI- matched premenopausal women (Table 3).

Multiple linear regression analyses showed that menopausal status was an independent determinant of total cholesterol after adjustment for age, BMI, systolic blood pressure, and fasting glucose (β = 0.08, $P < 0.05$) in the total number of subjects (Table 4). Menopausal status was also an independent determinant of HbA1c after adjustment for age, BMI, systolic blood pressure, and total cholesterol (β = 0.11, $P < 0.01$) in the total number of subjects (Table 5).

Discussion

Using data obtained from the KNHANES from 2016 to 2018, we demonstrated that menopausal status was associated with glucose and cholesterol levels, independent of age and BMI, among middle-aged Korean women. Multiple linear regression analyses showed that menopausal status was an independent determinant of total cholesterol and HbA1c. Menopausal status showed a significant relationship with increased total cholesterol levels even after adjusting for age and BMI in non-obese women, not obese women.

The effects of menopause on the lipid profiles during the menopausal transition in our study were comparable with the results of previous studies. Numerous epidemiological studies suggest menopause-associated changes in the lipid profile. In the Study of Women's Health Across the Nation, which was a longitudinal, community-based, multiethnic population study, total cholesterol and LDL cholesterol levels increased significantly within a year of the final menstrual period in 1,054 women who achieved the final menstrual period by the end of 9 years of follow-up during the menopausal transition [10]. In

middle-aged Caucasian women (age: 47-55 years), the menopausal transition was associated with increased total cholesterol, LDL cholesterol, and HDL cholesterol levels, independent of age [17]. In 593 healthy Chinese women aged 35 to 64 years, late perimenopausal status showed a significant association with an accelerated increase in total cholesterol and triglycerides. However, HDL cholesterol levels did not differ among the different menopausal status groups [9]. Of the 1,169 perimenopausal Korean women (age: 40-64 years) from the KNHANES 2005, postmenopausal women exhibited higher total cholesterol and LDL cholesterol levels than premenopausal women. However, there were no significant differences in HDL cholesterol levels between premenopausal and postmenopausal women after excluding subjects on medications for hypercholesterolemia [8]. Consistent with the results of our study, total cholesterol level was higher in postmenopausal women than premenopausal women, mostly in previous studies. Although we could not clarify the mechanism of change in lipid profiles during the menopausal transition in this study, decreasing estrogen levels during the menopausal transition known to affect hepatic lipase and lipoprotein lipase activity [18-20] may have an important role in the lipid metabolism during the menopausal transition. However, HDL and LDL cholesterol results according to the menopausal status were inconsistent between studies. We estimated LDL cholesterol using the Friedewald equation, contrary to the study on Caucasian women [17]. The age range of the participants was narrow in our study compared to previous studies on Chinese [9] or Korean women [8]. The age of the study participants, differences in methods measuring cholesterol levels, or different ethnicities may be responsible for the differences in LDL and HDL cholesterol results in women according to the menopausal status between studies.

The metabolic syndrome is characterized by abdominal adiposity, insulin resistance, and dyslipidemia and is closely associated with cardiovascular diseases [21]. The prevalence of metabolic syndrome increased during the menopausal transition in 949 women without diabetes, or the metabolic syndrome at baseline was independent of aging in the Study of Women’s Health Across the Nation [22]. Postmenopausal status was associated with an increased risk of metabolic syndrome after adjusting for age in 2,671 women who did not receive hormone replacement therapy in the KNHANES 2001 [23].

Postmenopausal status was an independent risk factor for metabolic syndrome after adjustment for age and BMI in 1,002 Korean women who participated in annual health examinations [24]. Postmenopausal status was associated with dysglycemia independent of aging in Japanese individuals [25]. A prospective study including 1,303 British women (age: 53 years) showed that HbA1c levels increased across the natural menopause transition after adjustment for BMI [26]. In a cross-sectional study, postmenopausal women showed higher HbA1c levels than premenopausal women after adjustments for age and BMI in Chinese women with BMI < 30 kg/m^2 [27]. HbA1c estimates long-term glucose status and is a better predictor of cardiovascular disease than fasting or post-challenge glucose in women without diabetes mellitus [28]. Consistent with the results of previous studies, postmenopausal women had higher HbA1c levels than premenopausal women in our subjects after adjustment for age and BMI, and menopausal status was an independent determinant of HbA1c in multiple linear regression analyses. Although we did not estimate abdominal obesity indicators, such as waist circumference or waist circumference/hip ratio, abdominal obesity accompanying menopause may be associated with increased insulin resistance and glucose levels in postmenopausal women [29].

In a study using the database from the KNHANES 2007-2010, waist circumference was significantly associated with systolic blood pressure after adjustment for age and BMI in 1,422 women (age: 45-55 years) during the menopausal transition. Waist circumference was associated with systolic blood pressure in non-obese women but not in obese women in this study group [30]. A cross-sectional study conducted in health-screening centers involving 2,037 Korean women (age: 44-56 years) showed significantly higher systolic blood pressure and diastolic blood pressure values during the late (than early) menopausal transition [11]. Blood pressure depended more on age than the menopausal status among the middle-aged Korean women from the KNHANES 2005, after excluding women on medication for hypertension [8]. Although we did not evaluate the blood pressure between late and early menopausal transition or the association between waist circumference and blood pressure among women during the menopausal transition, blood pressure did not differ between postmenopausal and

premenopausal women after adjustment for age and BMI in our study, consistent with the result of the previous KNHANES 2005.

The effect of menopause on cardiovascular risk factors differed according to BMI among middle-aged Korean women in our study. Among 2,659 women followed in the Study of Women's Health Across the Nation annually for up to 7 years, both LDL cholesterol and total cholesterol peaked in late peri- and early menopause; however, increases in LDL cholesterol and total cholesterol were smallest in the highest baseline weight tertile [31]. A cross-sectional study involving 1,553 Korean women (age: 44-56 years) who underwent a health screening examination showed that an increased prevalence of high non-HDL cholesterol was associated with postmenopausal status and more pronounced in lean women than in overweight or obese women after excluding for subjects on lipid-lowering medications or history of hypercholesterolemia [32]. Studies show that the effect of BMI on serum reproductive hormone levels varies by menopausal status. Increasing BMI was associated with decreasing estradiol levels in premenopausal women and increasing estradiol levels in postmenopausal women among 3,257 participants in the Study of Women's Health Across the Nation [13]. Relatively high estradiol levels in postmenopausal women with higher BMI may affect the cardiovascular risk factors differently when compared to non-obese postmenopausal women. Further studies measuring levels of reproductive hormones concurrently to evaluate the mechanism of the BMI effect on cardiovascular risk factors during the menopausal transition are needed.

This study is the first to evaluate the effect of menopause on cardiovascular risk factors according to BMI in middle-aged Korean women during the menopausal transition. The strengths of our study include a database from a nationally representative survey. Furthermore, we used well-matched age and BMI postmenopausal and premenopausal women, which enabled us to perform group comparisons without bias.

There were several limitations in this study. First, because the study was a retrospective and observational study, we could not identify a cause-and-effect relationship or mechanism underlying the

changes in cardiovascular risk factors during the menopausal transition. Although we had adjusted for potential confounding factors such as age and BMI, residual confounding factors, such as smoking, alcohol intake, physical activity, or dietary habits, may have influenced the results of our study. The exclusion of subjects with missing data may have introduced a selection bias. We identified the menopausal status based on self-reports, and therefore, misclassification of menopausal status may have occurred. We did not exclude women on hormone replacement (which could affect the lipid parameters). Although BMI is the standard measure to define obesity, it does not represent body fat composition. We did not evaluate the body fat distribution, known to be affected by menopausal status.

In conclusion, intensive monitoring and treating lipid status, including lifestyle modification education, would be needed to prevent cardiovascular events during the transition to menopause, especially in non-obese subjects.

References

1. Roth GA, Johnson C, Abajobir A, Abd-Allah F, Abera SF, Abyu G, et al. Global, regional, and national burden of cardiovascular diseases for 10 causes, 1990 to 2015. J Am Coll Cardiol. 2017;70(1):1-25.

2. Carr MC. The emergence of the metabolic syndrome with menopause. J Clin Endocrinol Metab. 2003;88(6):2404-11.

3. Organization WH. Research on the menopause in the 1990s: report of a WHO scientific group. 1996.

4. El Khoudary SR, Greendale G, Crawford SL, Avis NE, Brooks MM, Thurston RC, et al. The menopause transition and women's health at midlife: a progress report from the Study of Women's Health Across the Nation (SWAN). Menopause. 2019;26(10):1213-27.

5. Kannel WB, Hjortland MC, McNAMARA PM, Gordon T. Menopause and risk of cardiovascular disease: the Framingham study. Ann Intern Med. 1976;85(4):447-52.

6. Gordon T, KANNEL WB, HJORTLAND MC, McNAMARA PM. Menopause and coronary heart disease: the Framingham Study. Ann Intern Med. 1978;89(2):157-61.

7. El Khoudary SR, Aggarwal B, Beckie TM, Hodis HN, Johnson AE, Langer RD, et al. Menopause transition and cardiovascular disease risk: implications for timing of early prevention: a scientific statement from the American Heart Association. Circulation. 2020;142(25):e506-32.

8. Park HA, Park JK, Park SA, Lee JS. Age, menopause, and cardiovascular risk factors among Korean middle-aged women: the 2005 Korea National Health and Nutrition Examination Survey. J Womens Health. 2010;19(5):869-76.

9. Zhou J-L, Lin S-Q, Shen Y, Chen Y, Zhang Y, Chen F-L. Serum lipid profile changes during the menopausal transition in Chinese women: a community-based cohort study. Menopause. 2010;17(5):997-1003.

10. Matthews KA, Crawford SL, Chae CU, Everson-Rose SA, Sowers MF, Sternfeld B, et al. Are

changes in cardiovascular disease risk factors in midlife women due to chronological aging or to the menopausal transition? J Am Coll Cardiol. 2009;54(25):2366-73.

11. Son MK, Lim N-K, Lim J-Y, Cho J, Chang Y, Ryu S, et al. Difference in blood pressure between early and late menopausal transition was significant in healthy Korean women. BMC Womens Health. 2015;15(1):1-7.

12. Koo BK, Park S-H, Han K, Moon MK. Cardiovascular Outcomes of Obesity According to Menopausal Status: A Nationwide Population-Based Study. Endocrinol Metab. 2021;36(5): 1029-41.

13. Randolph Jr JF, Sowers M, Bondarenko IV, Harlow SnD, Luborsky JL, Little RJ. Change in estradiol and follicle-stimulating hormone across the early menopausal transition: effects of ethnicity and age. J Clin Endocrinol Metab. 2004;89(4):1555-61.

14. Kweon S, Kim Y, Jang M-j, Kim Y, Kim K, Choi S, et al. Data resource profile: the Korea national health and nutrition examination survey (KNHANES). Int J Epidemiol. 2014;43(1):69-77.

15. Region WWP. International Association for the Study of Obesity, and International Obesity Taskforce: The Asian-Pacific Perspective: Redefining Obesity and Its Treatment. Geneva, Switzerland, WHO Western Pacific Region. 2000.

16. Friedewald WT, Levy RI, Fredrickson DS. Estimation of the concentration of low-density lipoprotein cholesterol in plasma, without use of the preparative ultracentrifuge. Clin Chem. 1972;18(6):499-502.

17. Karvinen S, Jergenson MJ, Hyvärinen M, Aukee P, Tammelin T, Sipilä S, et al. Menopausal status and physical activity are independently associated with cardiovascular risk factors of healthy middle-aged women: cross-sectional and longitudinal evidence. Front Endocrinol. 2019;10:589.

18. Berg GA, Siseles N, González AI, Ortiz OC, Tempone A, Wikinski RW. Higher values of hepatic lipase activity in postmenopause: relationship with atherogenic intermediate density

and low density lipoproteins. Menopause. 2001;8(1):51-57.

19. Basdevant A, de Lignieres B, Simon P, Blache D, Ponsin G, Guy-Grand B. Hepatic lipase activity during oral and parenteral 17β-estradiol replacement therapy: high-density lipoprotein increase may not be antiatherogenic. Fertil Steril. 1991;55(6):1112-7.

20. Ferrara CM, Lynch NA, Nicklas BJ, Ryan AS, Berman DM. Differences in adipose tissue metabolism between postmenopausal and perimenopausal women. J Clin Endocrinol Metab. 2002;87(9):4166-70.

21. Isomaa B, Almgren P, Tuomi T, Forsén B, Lahti K, Nissén M, et al. Cardiovascular morbidity and mortality associated with the metabolic syndrome. Diabetes care. 2001;24(4):683-9.

22. Janssen I, Powell LH, Crawford S, Lasley B, Sutton-Tyrrell K. Menopause and the metabolic syndrome: the Study of Women's Health Across the Nation. Arch Intern Med. 2008;168(14):1568-75.

23. Kim HM, Park J, Ryu SY, Kim J. The effect of menopause on the metabolic syndrome among Korean women: the Korean National Health and Nutrition Examination Survey, 2001. Diabetes care. 2007;30(3):701-6.

24. Cho GJ, Lee JH, Park HT, Shin JH, Hong SC, Kim T, et al. Postmenopausal status according to years since menopause as an independent risk factor for the metabolic syndrome. Menopause. 2008;15(3):524-9.

25. Heianza Y, Arase Y, Kodama S, Hsieh SD, Tsuji H, Saito K, et al. Effect of postmenopausal status and age at menopause on type 2 diabetes and prediabetes in Japanese individuals: Toranomon Hospital Health Management Center Study 17 (TOPICS 17). Diabetes care. 2013;36(12):4007-14.

26. Kuh D, Langenberg C, Hardy R, Kok H, Cooper R, Butterworth S, et al. Cardiovascular risk at age 53 years in relation to the menopause transition and use of hormone replacement therapy: a prospective British birth cohort study. BJOG. 2005;112(4):476-85.

27. Chang C-J, Wu C-H, Yao W-J, Yang Y-C, Wu J-S, Lu F-H. Relationships of age, menopause

and central obesity on cardiovascular disease risk factors in Chinese women. Int J Obes Relat Metab Disord. 2000;24(12):1699-704.

28. Park S, Barrett-Connor E, Wingard DL, Shan J, Edelstein S. GHb is a better predictor of cardiovascular disease than fasting or postchallenge plasma glucose in women without diabetes: the Rancho Bernardo Study. Diabetes care. 1996;19(5):450-6.

29. Pouliot M, Després J, Nadeau A, Moorjani S, Prud'Homme D, Lupien P, et al. Visceral Obesity in Men: Associations With Glucose Tolerance, Plasma Insulin, and Lipoprotein Levels. Diabetes. 1992;41(7):826-34.

30. Park JK, Lim Y-H, Kim K-S, Kim SG, Kim JH, Lim HG, et al. Changes in body fat distribution through menopause increase blood pressure independently of total body fat in middle-aged women: the Korean National Health and Nutrition Examination Survey 2007–2010. Hypertens Res. 2013;36(5):444-9.

31. Derby CA, Crawford SL, Pasternak RC, Sowers M, Sternfeld B, Matthews KA. Lipid changes during the menopause transition in relation to age and weight: the Study of Women's Health Across the Nation. Am J Epidemiol. 2009;169(11):1352-61.

32. Choi Y, Chang Y, Kim B-K, Kang D, Kwon M-J, Kim C-W, et al. Menopausal stages and serum lipid and lipoprotein abnormalities in middle-aged women. Maturitas. 2015;80(4):399-405.

Table 1. Baseline Characteristics of Study Participants

	Total subjects			
	Premenopausal women (n = 758)	Postmenopausal women (n = 361)	*P*-value	Adjusted *P*-value*
Age (y)	48 ± 2	52 ± 2	<0.001	
BMI (kg/m^2)	23.0 ± 3.1	22.8 ± 2.9	0.395	
Fasting glucose (mg/dL)	93 ± 12	94 ± 9	0.241	0.122
HbA1c (%)	5.4 ± 0.4	5.5 ± 0.3	<0.001	0.002
Total cholesterol (mg/dL)	191 ± 28	201 ± 25	<0.001	0.045
HDL cholesterol (mg/dL)	55 ± 12	56 ± 13	0.366	0.650
Triglycerides (mg/dL)	96 ± 56	101 ± 48	0.190	0.499
LDL cholesterol (mg/dL)	116 ± 25	125 ± 22	<0.001	0.087
Systolic BP (mmHg)	110 ± 11	110 ± 12	0.209	0.185
Diastolic BP (mmHg)	73 ± 8	73 ± 8	0.214	0.977

Plus-minus values are the means ± the standard deviations.

* Adjusted for age and body mass index.

BMI, body mass index; HbA1c, hemoglobin A1c; HDL cholesterol, high-density lipoprotein cholesterol; LDL cholesterol; low-density lipoprotein cholesterol; BP, blood pressure.

Table 2. Comparison of Characteristics and Cardiovascular Risk Factors in Study Participants According to the Degree of Obesity

	Non-obese subjects				Obese subjects			
	Premenopausal women (n = 575)	Postmenopausal women (n = 291)	*P*-value	Adjusted *P*-value*	Premenopausal women (n = 183)	Postmenopausal women (n = 70)	*P*-value	Adjusted *P*-value*
Age (y)	48 ± 3	52 ± 2	<0.001		48 ± 2	53 ± 2	<0.001	
BMI (kg/m^2)	21.6 ± 1.9	21.7 ± 1.8	0.322		27.3 ± 2.1	27.3 ± 2.2	0.996	
Fasting glucose (mg/dL)	92 ± 8	93 ± 8	0.013	0.091	97 ± 18	97 ± 11	0.776	0.580
HbA1c (%)	5.4 ± 0.3	5.5 ± 0.3	<0.001	0.001	5.5 ± 0.5	5.6 ± 0.3	0.512	0.483
Total cholesterol (mg/dL)	190 ± 27	201 ± 25	<0.001	0.032	194 ± 28	201 ± 24	0.045	0.966
HDL cholesterol (mg/dL)	57 ± 12	58 ± 12	0.506	0.185	51 ± 11	50 ± 11	0.848	0.064
Triglycerides (mg/dL)	90 ± 42	95 ± 42	0.066	0.549	117 ± 82	123 ± 64	0.584	0.669
LDL cholesterol (mg/dL)	115 ± 24	124 ± 22	<0.001	0.123	120 ± 26	126 ± 23	0.061	0.575
Systolic BP (mmHg)	108 ± 11	110 ± 12	0.155	0.258	113 ± 11	114 ± 13	0.481	0.547
Diastolic BP (mmHg)	72 ± 7	73 ± 7	0.210	0.989	75 ± 7	76 ± 8	0.369	0.855

Plus-minus values are the means ± the standard deviations.

* Adjusted for age and body mass index.

BMI, body mass index; HbA1c, hemoglobin A1c; HDL cholesterol, high-density lipoprotein cholesterol; LDL cholesterol; low-density lipoprotein cholesterol; BP, blood pressure.

Table 3. Comparison of Characteristics and Cardiovascular Risk Factors in Age- and BMI-Matched Participants

	Total subjects			Non-obese subjects			Obese subjects		
	Premenopausal women (n = 138)	Postmenopausal women (n = 138)	*P*-value	Premenopausal women (n = 117)	Postmenopausal women (n = 117)	*P*-value	Premenopausal women (n = 21)	Postmenopausal women (n = 21)	*P*-value
Age (y)	51 ± 2	51 ± 2	1.000	51 ± 2	51 ± 2	1.000	51 ± 2	51 ± 2	1.000
BMI (kg/m^2)	22.5 ± 2.4	22.6 ± 2.4	0.792	21.7 ± 1.6	21.8 ± 1.6	0.653	26.7 ± 1.8	26.7 ± 1.7	0.960
Fasting glucose (mg/dL)	93 ± 9	93 ± 7	0.710	93 ± 9	93 ± 7	0.836	95 ± 8	96 ± 7	0.602
HbA1c (%)	5.5 ± 0.3	5.5 ± 0.3	0.099	5.5 ± 0.4	5.5 ± 0.3	0.152	5.4 ± 0.3	5.5 ± 0.3	0.381
Total cholesterol (mg/dL)	196 ± 27	201 ± 25	0.091	194 ± 25	201 ± 25	0.060	205 ± 36	206 ± 23	0.944
HDL cholesterol (mg/dL)	56 ± 11	59 ± 13	0.116	56 ± 11	59 ± 14	0.051	56 ± 9	53 ± 10	0.313
Triglycerides (mg/dL)	93 ± 39	99 ± 46	0.246	90 ± 37	96 ± 44	0.290	109 ± 43	118 ± 50	0.575
LDL cholesterol (mg/dL)	122 ± 23	123 ± 21	0.497	120 ± 22	122 ± 21	0.508	127 ± 31	129 ± 21	0.815
Systolic BP (mmHg)	110 ± 11	108 ± 12	0.387	109 ± 11	109 ± 12	0.808	114 ± 11	108 ± 12	0.106
Diastolic BP (mmHg)	73 ± 7	73 ± 8	0.527	72 ± 7	73 ± 7	0.318	76 ± 5	74 ± 9	0.490

Plus-minus values are the means ± the standard deviations.

BMI, body mass index; HbA1c, hemoglobin A1c; HDL cholesterol, high-density lipoprotein cholesterol; LDL cholesterol; low-density lipoprotein cholesterol; BP, blood pressure.

Table 4. Association of Menopausal Status with Total Cholesterol by Multiple Linear Regression Analyses

Variable	Unstandardized coefficients		Standardized coefficients	*P*-value	CI
	β	SE	β		
Menopausal status-unadjusted	10.47	1.73	0.18	<0.001	7.08-13.87
BMI-adjusted	10.59	1.72	0.18	<0.001	7.21-13.97
Age, BMI-adjusted	4.48	2.23	0.08	0.045	0.11-8.85
Age, BMI, SBP-adjusted	4.59	2.23	0.08	0.040	0.21-8.96
Age, BMI, SBP, fasting glucose-adjusted	4.58	2.23	0.08	0.041	0.20-8.96

SE, standard error; CI, confidence interval; BMI, body mass index; SBP, systolic blood pressure.

Table 5. Association of Menopausal Status with Hemoglobin A1c by Multiple Linear Regression Analyses

Variable	Unstandardized coefficients		Standardized coefficients	*P*-value	CI
	β	SE	β		
Menopausal status-unadjusted	0.12	0.02	0.16	<0.001	0.08-0.17
BMI-adjusted	0.13	0.02	0.17	<0.001	0.08-0.17
Age, BMI-adjusted	0.09	0.03	0.12	0.002	0.03-0.14
Age, BMI, SBP-adjusted	0.09	0.03	0.12	0.002	0.03-0.15
Age, BMI, SBP, total cholesterol-adjusted	0.08	0.03	0.11	0.004	0.03-0.14

SE, standard error; CI, confidence interval; BMI, body mass index; SBP, systolic blood pressure.
